# Correlation of Serum C-Peptide, Soluble Intercellular Adhesion Molecule-1, and NLRP3 Inflammasome-Related Inflammatory Factor Interleukin-1*β* after Brain Magnetic Resonance Imaging Examination with Cerebral Small Vessel Disease

**DOI:** 10.1155/2022/4379847

**Published:** 2022-01-27

**Authors:** Chunli Ma, Lei Yang, Lihua Wang

**Affiliations:** ^1^The Second Clinical Medical College of Harbin Medical University, Harbin 150000, Heilongjiang, China; ^2^Department of Neurology, The Second Affiliated Hospital of Mudanjiang Medical College, Mudanjiang 157000, Heilongjiang, China; ^3^Clinical Skills Training Center, First Clinical Medical College, Mudanjiang Medical College, Mudanjiang 157011, Heilongjiang, China; ^4^Neurology Fourth Ward, The 2nd Affiliated Hospital of Harbin Medical University, Harbin 150001, Heilongjiang, China

## Abstract

**Objective:**

To explore the correlation of serum c-peptide, soluble intercellular adhesion molecule-1 (sICAM-1), and NLRP3 inflammasome-related inflammatory factor interleukin-1*β* (IL-1*β*) after brain magnetic resonance imaging (MRI) examination with cerebral small vessel disease (CSVD).

**Methods:**

A total of 72 CSVD patients treated in our hospital from December 2018 to December 2019 were selected as the case group and another 72 patients who presented cerebrovascular risk factors but obtained normal brain MRI examination result in the same period were selected as the control group. The serum specimen of patients in the two groups were collected, their serum c-peptide levels were measured by radio immunoassay, and their serum sICAM-1 and NLRP3 inflammasome-related inflammatory factor IL-1*β* were measured by enzyme-linked immunosorbent assay (ELISA), so as to analyze the correlation between these indicators and CSVD.

**Results:**

Compared with the control group, the level values of serum c-peptide, sICAM-1, and IL-1*β* were significantly higher in the case group (*P* < 0.001), with CSVD being the dependent variable, and age, smoking, uric acid, history of stroke, serum c-peptide, sICAM-1, and IL-1*β* being the independent variables. A logistic regression analysis was conducted, and the result showed that age, smoking, serum c-peptide, sICAM-1, and IL-1*β* were the risk factors for CSVD, and by drawing the ROC curves, it could be concluded that the area under sICAM-1 curve was larger than that of other single indicator.

**Conclusion:**

Elevation of level values of serum c-peptide, sICAM-1, and NLRP3 inflammasome-related inflammatory factor IL-1*β* is correlative with CSVD, and age, smoking, serum c-peptide, sICAM-1, and IL-1*β* are the independent risk factors for CSVD.

## 1. Introduction

Cerebral small vessel disease (CSVD) refers to the disease caused by small vessel lesion in the brain, which involves a vessel diameter of 30–800 *μ*m [1, 2]. It is a brain wide dynamic disease, the exact pathogenesis of which has not been fully elucidated, and it is currently believed to be mainly related to hypoperfusion, microembolic embolism, ischemic damage, structural changes in small vessels, and some metabolic encephalopathies, such as homocysteinemia. With the acceleration of population aging and high frequency of risk factors for cerebrovascular disease, the prevalence of CSVD is increasing year by year. Recent advances in science and technology have deepened the understanding of CSVD [3], and studies have shown that CSVD increases patients' risk of developing cerebral infarction and death. Meanwhile, further studies have confirmed that the impairment of CSVD on cognitive function increases patients' probability of developing dementia, undoubtedly illustrating the immense danger of the disease [4, 5]. CSVD has a complex cause of disease with variable and more insidious clinical symptoms, and its diagnosis mainly relies on imaging examinations, so early detection and implementation of targeted intervention is the key to improving the prognosis of the disease [6]. At present, age, hypertension, etc., are recognized risk factors for CSVD, but there is a lack of specific serological markers. Previous studies have confirmed that serum c-peptide can indirectly reflect the concentration of insulin, and insulin resistance is related to the abnormal increase of insulin content, which is an independent risk factor for the occurrence of CSVD [7]. NLRP3 inflammasome is an important pattern recognition receptor in the cytoplasm, which can sense pathogenic microorganisms and metabolites in cells and start the assembly of inflammatory complex. As an inflammatory factor of NLRP3, IL-1*β* is one of the key messengers involved in the interaction between the CNS and the immune system, and it also involves in immune regulation in the CNS and promotes neuronal growth and development under physiological conditions, but its high concentrations will inhibit the excitability and synaptic function of neurons in the hippocampal region, which in turn will lead to cognitive dysfunction [8]. Soluble intercellular adhesion molecule-1 (sICAM-1), as a biomarker of endothelial cell injury, participates in inflammatory response. Some studies [9] have found that a higher level of sICAM-1 may be a risk factor for leukoaraiosis. Leukoaraiosis refers to the hypersoftening of cerebral white matter, which is a normal aging process, but will lead to leukoaraiosis when softening is too fast. Currently, there are no reports pointing out the correlation of serum c-peptide, NLRP3 inflammasome-related inflammatory factor IL-1*β*, and sICAM-1 after brain MRI examination with CSVD. To prove the aforesaid opinions, the study was carried out, and the results are reported as follows.

## 2. Materials and Methods

### 2.1. General Data

A total of 72 CSVD patients treated in our hospital from December 2018 to December 2019 were selected as the case group, and 72 patients who presented cerebrovascular risk factors but obtained normal brain MRI examination result in the same period were selected as the control group. The study met the *World Medical Association Declaration of Helsinki (2013*) [10]. Within 24 after admission, neurologists collected the clinical data and information of the study subjects who met the enrollment conditions, including their gender, age, smoking history, history of hypertension, and total cholesterol (TC) level.

### 2.2. Inclusion and Exclusion Criteria

Inclusion criteria: ① the patients met the *Diagnostic Criteria for CSVD Revised by National Conference on Cerebrovascular Diseas*es [11] and were aged between 18 and 80 years; ② the patients had focal neurological signs; ③ the patients had an acute or subacute onset; and ④ the patients did not have history of serious brain injury.

Exclusion criteria: ① the patients had stroke with other specific causes; ② the patients were unconscious or were unable to cooperate with the study; ③ the patients were complicated with severe dysfunction of kidney, liver, lung, etc.; and ④ the patients presented obvious intracranial artery atherosclerosis.

### 2.3. Methods

Patients in the two groups received the brain MRI examination for measurement of their expression levels of serum c-peptide, NLRP3 inflammasome-related inflammatory factor IL-1*β*, and sICAM-1, with the following specific methods.

Brain MRI examination: Philip 1.5T MRI system (manufactured: Koninklijke Philips N.V.) was used for brain scan in the conventional axial position and sagittal positions T1WI (TR 550 ms, TE 15 ms, slice thickness 4 mm, slice gap 0.4 mm, and 20 slices), T2WI (TR 2.7 ms, TE 150 ms, slice thickness 4 mm, slice gap 0.4 mm, and 20 slices), and DWI (TR 2.7 ms, TE 50 ms, *b* value 1,000 s/mm^2^, slice thickness 4 mm, slice gap 0.4 mm, and 20 slices); after image collection, two senior imaging physicians analyzed the images by the double blind method to observe and record situations such as leukoaraiosis and cerebral microhemorrhage.

Serum detection: the patients in the two groups were checked and told the objective and cooperation method of blood collection. 5 ml of fasting venous blood was drawn from their suitable vein in the morning and placed for 10–20 min under room temperature and then centrifuged under 3,000 r/min for 15 min to collect the supernatant and put it in an ultra-low temperature freezer. In case of precipitation occurred during preservation, the blood samples should be centrifuged again for supernatant collection. The serum c-peptide level was measured by the radioimmunoassay with the kits purchased from Nanjing Xinfan Biotechnology Co., Ltd. The IL-1*β* and sICAM-1 levels were measured by the ELISA method with the experiment steps in strict accordance with the specification on the kits (manufactured: Wuhan Adanti Biotechnology Co., Ltd.). The kits were required to be preserved under the environment of 2–8°C before use; if use, the kits should be taken out from the cold storage environment and then placed under the room temperature for 15–30 min before operation.

### 2.4. Statistical Methods

In this study, the data were processed with the professional statistical software SPSS23.0, and the picture drawing software was GraphPad Prism 7 (GraphPad Software, San Diego, USA). The related risk factors for the occurrence of CSVD were analyzed by multifactor logistic regression, and differences were considered statistically significant at *P* < 0.05.

## 3. Results

### 3.1. Comparison of Clinical Data between the Case Group and the Control Group

Other than the mean age, smoking history, uric acid, and history of stroke, other clinical indicators were not significantly different between the two groups (*P* > 0.05). See Table 1.

### 3.2. Between-Group Comparison of Level Values of Serum C-Peptide, sICAM-1, and IL-1*β*

Compared with the control group, the level values of serum c-peptide, sICAM-1, and IL-1*β* were significantly higher in the case group (*P* < 0.001). See Table 2.

### 3.3. Correlation of Serum C-peptide, sICAM-1, and IL-1*β* with CSVD

With CSVD being the dependent variable, and age, smoking, uric acid, history of stroke, serum c-peptide, sICAM-1, and IL-1*β* being the independent variables, a logistic regression analysis was conducted, and the result showed that age, smoking, serum c-peptide, sICAM-1, and IL-1*β* were the risk factors for CSVD. See Table 3.

### 3.4. ROC Curve Analysis on Serum C-Peptide, sICAM-1, IL-1*β*, and CSVD

**Figure 1:**
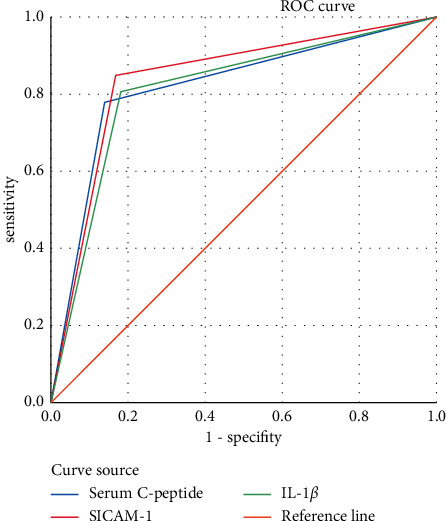
ROC curve analysis on serum c-peptide, sICAM-1, IL-1*β*, and CSVD.

Figure 1 shows the ROC curve analysis on serum c-peptide, sICAM-1, IL-1*β*, and CSVD.

### 3.5. Comparison of Areas under the Curves among Various Indicators

The area under the curve of sICAM-1 was larger than that under other single indicator. See Table 4.

## 4. Discussion

As the population aging deepens in recent years, the prevalence of senile dementia is increasing, which not only puts great pressure on society and families but also seriously affects the physical and mental health of the elderly population, making it an important cause of death in them [12]. CSVD is an intracranial small vessel lesion in sites such as arterioles, small veins, and capillaries caused by a combination of factors, which damages the white matter and gray matter in depth of brain and finally leads to a series of pathological and neuroimaging changes, thereby resulting in cognitive impairment and other clinical syndromes in patients [13]. Studies have confirmed [14] that CSVD is not only related to vascular cognitive dysfunction but also to the pathogenesis of neurodegenerative diseases such as Alzheimer's disease (AD), and as these two diseases are overlapped to some extent, most AD patients have signs of CSVD, including cognitive dementia, progressive cognitive decline, bladder sphincter dysfunction, and mental disorders [15]. Currently, there is no consensus on CSVD pathogenesis, but it may be due to the fact that chronic progressive subclinical ischemia in the brain increases vascular permeability, causing extravasation of intravascular substances, disruption of the blood-brain barrier, local inflammatory response, and then resulting in vascular and perivascular tissue damage [16].

Brain MRI has become a common imaging modality at present, and it applies radiofrequency pulses of specific frequency to the human body in the static magnetic field, so that the hydrogen protons in the human body are stimulated and then cause magnetic resonance; after stopping the pulse, the protons produce MR signal during relaxation, and with the continuous promotion of MRI imaging sequence and image, brain MRI provides a good basis for the diagnosis of brain lesions [17]. Moreover, MRI is effective in detecting cerebral small vessel injury because it is noninvasive and easy to operate. However, applying MRI for early screening of CSVD is more costly and time-consuming, so serologic markers have become a new research direction.

A foreign meta-analysis found that [18] compared with nonstroke patients, CSVD patients have significantly increased serum c-reactive protein (CRP), tumor necrosis factor-*α* (TNF-*α*), and interleukin-6 (IL-6), indicating that the inflammatory process participates in the pathomechanism of CSVD. The activation and amplification of inflammatory response is an important factor in the development and progression of inflammatory diseases, and the immune inflammasome is the initiator of inflammation. Current studies have confirmed that [19] the increased expression level of NLRP3 inflammasome is closely related to the occurrence and progression of chronic obstructive pulmonary disease, malignancy, and chronic viral hepatitis. As a macromolecular multiprotein complex, NLRP3 inflammasome functions to regulate the secretion and maturation of downstream proinflammatory cytokines such as IL-8 and IL-1*β*. These proinflammatory cytokines produced by the activated NLRP3 inflammasome can mediate the activation of the NF-kB signaling pathway and induce the occurrence of brain neuroinflammation. When cerebral ischemia occurs, IL-1*β* and other inflammatory mediators can activate local endothelial cells and leukocytes and induce significant upregulation of the number of adhesion molecules on the cell surface, which leads to massive adhesion between leukocytes and endothelial cells, causing microvascular obstruction and aggravating brain tissue damage [20, 21]. High insulin level is a well-established risk factor for atherosclerosis, whereas microvascular endothelial cells are vulnerable to insulin metabolism and growth promoting effects. Serum c-peptide can indirectly reflect the concentration of insulin, and abnormally increased insulin content together with insulin resistance is an independent risk factor contributing to the development of CSVD [22]. SICAM-1 is an inflammatory factor secreted by vascular endothelial cells that mediate adhesion of platelets, leukocytes, and vascular endothelium and is involved in blood-brain barrier (BBB) disruption and brain tissue damage induced by chronic cerebral ischemia, and high levels of sICAM-1 may also increase the risk of future recurrent stroke and vascular dementia [23].

The study found that compared with the control group, the level values of serum c-peptide, sICAM-1, and IL-1*β* were significantly higher in the case group, implying that the level values of these serological markers were related to the development of CSVD. The study by Saadi Mahdiye et al. [8] found that the elevated sICAM-1 and osteoprotegerin are the independent risk factors for leukoaraiosis, and another meta-analysis found that [24] c-peptide levels cause endothelial function lesions in cerebral small vessel and are one of the main causes of cerebral white matter hyperintensity. The white matter is a normal component of the brain, generally concentrated in the centrum semiovale and basal ganglia and is the portion of neuronal axons and myelin that is also subject to neurological dysfunction when damaged because it is involved in nerve conduction. Further logistic regression analysis concluded that age, smoking, serum c-peptide, sICAM-1, and IL-1*β* were the risk factors for CSVD, which was of great importance to clinical treatment and prognosis. Limitations of the study: as a single-center study, the results only represented the disease characteristics of a certain region or group; and the case group was not subdivided into two subgroups (with or without acute cerebral infarction) for intercomparison.

## Figures and Tables

**Table 1:** Between-group comparison of clinical data.

Item	Case group	Control group	*X* ^2^/*t*	*P*
Gender			0.251	0.616
Male/female	37/35	40/32		
BMI (mean ± SD, kg/m^2^)	21.23 ± 1.72	21.63 ± 1.94	1.309	0.193
Mean age (mean ± SD, years)	64.68 ± 8.23	54.60 ± 5.73	8.529	<0.001
Smoking history			28.466	<0.001
Yes	53 (73.61%)	21 (29.17%)		
No	19 (26.39%)	51 (70.83%)		
Drinking history			0.113	0.736
Yes	40 (55.56%)	42 (58.33%)		
No	32 (44.44%)	30 (41.67%)		
History of hypertension			0.256	0.613
Yes	40 (55.56%)	43 (59.72%)		
No	32 (44.44%)	29 (40.28%)		
History of coronary heart disease			0.028	0.867
Yes	40 (55.56%)	39 (54.17%)		
No	32 (44.44%)	33 (45.83%)		
Fasting blood glucose value (mean ± SD, mmol/L)	5.42 ± 1.09	5.28 ± 1.17	0.743	0.459
TC (mean ± SD, mmol/L)	4.28 ± 0.76	4.05 ± 0.83	1.734	0.085
Triacylglycerol (mean ± SD, mmol/L)	1.16 ± 0.41	1.19 ± 0.38	0.455	0.650
Uric acid (mean ± SD, *μ*mol/L)	324.64 ± 19.69	243.51 ± 12.51	29.510	<0.001
History of stroke			17.383	<0.001
Yes	20 (27.78%)	2 (2.78%)		
No	52 (72.22%)	70 (97.22%)		

**Table 2:** Between-group comparison of level values of serum c-peptide, sICAM-1, and IL-1*β* (mean ± SD).

Group	n	Serum c-peptide (pmol/L)	sICAM-1 (ng/ml)	IL-1*β* (ng·L^−1^)
Case group	72	1.54 ± 0.09	153.96 ± 4.90	21.16 ± 0.75
Control group	72	0.77 ± 0.14	101.37 ± 1.56	15.36 ± 1.29
t		39.257	87.778	33.323
P		＜0.001	＜0.001	＜0.001

**Table 3:** Multi-factor logistic regression analysis on CSVD.

Item	*B*	SE	Wald	*P*	OR
Age	0.168	0.073	0.016	0.006	1.253
Smoking	4.235	1.376	7.263	0.012	0.025
Uric acid	0.527	0.326	5.273	0.427	1.836
History of stroke	16.278	4.721	0.263	0.724	2.367
Serum c-peptide	0.753	0.352	5.261	0.025	2.031
sICAM-1	0.867	0.386	6.261	0.031	2.638
IL-1*β*	0.536	0.137	8.025	0.006	1.362

**Table 4:** Comparison of areas under the curves among various indicators.

Test result variable	Area	SE^a^	Asymp.sig.^b^	Asymp. 95% CI	
				Lower limit	Upper limit
Serum c-peptide	0.819	0.037	0.000	0.747	0.892
sICAM-1	0.840	0.035	0.000	0.771	0.910
IL-1*β*	0.813	0.038	0.000	0.739	0.886

## Data Availability

Data to support the findings of this study are available on reasonable request from the corresponding author.
